# Unmasking Suicidal Ideation for Asian American, Native Hawaiian, and Pacific Islander Youths Via Data Disaggregation

**DOI:** 10.1001/jamanetworkopen.2024.46832

**Published:** 2024-11-22

**Authors:** Camillia K. Lui, Yu Ye, Joyce Gee, Won Kim Cook, Christina C. Tam, Sicong Sun, Regina Miranda, Andrew Subica, Nina Mulia

**Affiliations:** 1 Alcohol Research Group, Public Health Institute, Emeryville, California; 2 Department of Social Welfare, Luskin School of Public Affairs, University of California, Los Angeles; 3 Department of Psychology, Hunter College, City University of New York, New York; 4 Department of Social Medicine, Population, and Public Health, University of California,, Riverside School of Medicine, Riverside

## Abstract

**Importance:**

Suicide is one of the leading causes of death for Asian American, Native Hawaiian, and Pacific Islander adolescents, but because these individuals are analyzed in aggregate, potentially important differences in suicide risk are obscured.

**Objective:**

To disaggregate Asian and Native Hawaiian or Pacific Islander adolescents into racial and ethnic subgroups and compare risks of suicidal ideation between and within the subgroups.

**Design, Setting, and Participants:**

This cross-sectional study used pooled data from 4-year public high school students (9th and 11th grade) participating in the 2017-2018 and 2018-2019 California Healthy Kids Survey. Data were analyzed between January 5, 2023, and March 31, 2024.

**Exposures:**

Self-reported race and ethnicity, including American Indian or Alaska Native, Asian, Black, Hispanic or Latinx, Native Hawaiian or Pacific Islander, and White, and 9 Asian and Native Hawaiian or Pacific Islander subgroups, including Asian Indian, Chinese, Filipino, Japanese, Korean, Native Hawaiian or Pacific Islander, other Asian, Southeast Asian (ie, Cambodian, Hmong, Laotian), and Vietnamese.

**Main Outcome and Measures:**

Past-year suicidal ideation was the primary outcome. Analyses compared an aggregated Asian and Native Hawaiian or Pacific Islander group with subgroups disaggregated by ethnicity and by monoethnic or monoracial, multiethnic, and multiracial identifications. Logistic regression models were used to test between- and within-group differences.

**Results:**

Among 547 816 California adolescents in 9th or 11th grade surveyed (50.3% girls), 77 735 (14.2%) identified as Asian or Native Hawaiian or Pacific Islander. The prevalence of suicidal ideation was 17.5% (95% CI, 17.1%-17.9%) among adolescents in the combined Asian and Native Hawaiian or Pacific Islander group. After disaggregation into subgroups, prevalence of suicidal ideation ranged from 13.3% (95% CI, 12.3%-14.4%) for monoethnic Asian Indian adolescents to 21.2% (95% CI, 20.4%-21.9%) for monoethnic Filipino adolescents. Multiethnic and multiracial identity was associated with elevated risk of suicidal ideation for all groups, from 2.3 percentage points (from 21.2% [95% CI, 20.4%-21.9%] among monoethnic to 23.5% [95% CI, 22.8%-24.2%] among multiracial Filipino adolescents) to 9.4 percentage points (from 13.7%, [95% CI, 13.0%-14.4%] among monoethnic to 23.1% [95% CI, 22.1%-24.1%] among multiracial Chinese adolescents). Compared with a mutually exclusive approach, an additive model allowing for self-report of multiple identities had the best fit and showed a higher prevalence of suicidal ideation among multiethnic and multiracial Asian and Native Hawaiian or Pacific Islander adolescents compared with monoethnic Chinese adolescents.

**Conclusions and Relevance:**

The results from this cross-sectional study provide evidence for disaggregation of heterogeneous ethnoracialized groups as a data equity strategy that may illuminate hidden disparities in adolescent suicidal ideation risk. Further research is imperative to understand suicide risk among Asian, Native Hawaiian, and Pacific Islander adolescents who identify with multiple ethnic or racial groups.

## Key Points

**Question:**

What is missed when the prevalence of adolescent suicidal ideation is aggregated among Asian American, Native Hawaiian, and Pacific Islander groups, and when disaggregated, which groups are at higher risk?

**Findings:**

In this cross-sectional study of 77 735 California adolescents who identified as Asian or Native Hawaiian or Pacific Islander, past-year suicidal ideation varied significantly among monoethnic Asian and Native Hawaiian or Pacific Islander (from 13.3% among Asian Indian to 21.2% among Filipino). Multiethnic and multiracial identity was associated with elevated risk of suicidal ideation for all groups, ranging from 2.3 percentage points higher for Filipino adolescents to 9.4 percentage points higher for Chinese adolescents.

**Meaning:**

These findings suggest that disaggregating heterogeneous ethnoracialized groups may provide data for meaningful prevention efforts to uncover hidden disparities in determining adolescent suicide risk.

## Teaser

This cross-sectional study compares risks of adolescent suicidal ideation between and within various Asian and Native Hawaiian or Pacific Islander racial and ethnic subgroups.

## Introduction

Suicide is one of the leading causes of death for Asian American, Native Hawaiian, and Pacific Islander adolescents in the US.^1^ Although they are distinct racialized groups,^2,3^ data on Asian and Native Hawaiian and Pacific Islander adolescents are often combined due to small sample sizes and systematic limitations in data collection.^4,5,6^ When examined separately, Asian, Native Hawaiian, and Pacific Islander adolescents exhibit similar or significantly higher suicidal behaviors compared with White adolescents.^7^ In the 2019 Youth Risk Behavioral Survey, 19.7% of Asian and 15.4% of Native Hawaiian or Pacific Islander adolescents reported seriously considering a suicide attempt compared with 16.9% of Black and 19.1% of White adolescents. Additionally, 7.7% of Asian and 8.8% of Native Hawaiian or Pacific Islander adolescents reported a past-year suicide attempt (compared with 11.8% of Black and 7.9% of White adolescents).^8^ Despite these comparable numbers, Asian, Native Hawaiian, and Pacific Islander adolescent suicide remains an invisible issue in research and funding. For example, a 2023 California request for applications for a youth suicide prevention program listed all ethnoracialized communities except for Asian, Native Hawaiian, and Pacific Islander communities. This decision was based on aggregated Asian, Native Hawaiian, and Pacific Islander death records that showed lower numbers and rates of youth suicide compared with other racially and ethnically minoritized communities. This aggregation of data may exacerbate the myth that these adolescents are immune to suicidal behaviors.

Researchers and community advocates have long called for more disaggregated research on Asian and Native Hawaiian or Pacific Islander populations,^9^ with recent studies pointing to inadequate funding and specificity in health research^10,11,12^ and limitations in population surveys and administrative health data.^13,14^ Importantly, data disaggregation is a crucial aspect of data equity for Asian, Native Hawaiian, and Pacific Islander people given their diverse needs across 50 ethnic groups comprising more than 30 countries of origin and more than 100 languages; different migration and US settlement experiences; and increasing numbers of individuals who identify as multiracial.^15,16^ Collecting and reporting this heterogeneity are necessary to develop culturally relevant interventions and antiracist policies to reduce suicide-related risks among Asian and Native Hawaiian or Pacific Islander adolescents.

The goals of this study were 2-fold. First, we examined adolescent suicide risks for 9 distinct Asian and Native Hawaiian or Pacific Islander subgroups to assess heterogeneity of risk. Going beyond a monoracial paradigm of race based on mutually exclusive categories,^17,18^ we considered monoethnic Asian, monoracial Native Hawaiian or Pacific Islander, multiethnic Asian (identification with >1 Asian ethnic subgroup), and multiracial Asian and Native Hawaiian or Pacific Islander (identification with >1 racialized group) identities. Risky health behaviors and outcomes have been documented among multiracial adolescents,^19^ but no study has examined multiethnic identifications. We expected suicidal ideation to vary greatly upon data disaggregation, particularly among multiracial adolescents. Second, as a methodological goal, we compared traditional aggregation and alternative disaggregation approaches in between- and within-group analyses to further identify suicide-related risks. We also examined the value of data disaggregation to identify public health needs of a heterogenous population that may otherwise be obscured.

## Methods

This cross-sectional study was deemed exempt from review and informed consent by the institutional review board of the Public Health Institute as non–human participant research. We followed the Strengthening the Reporting of Observational Studies in Epidemiology (STROBE) reporting guideline for cross-sectional studies.^20^

### Data Source

Data were pooled from the 2017-2018 and 2018-2019 California Healthy Kids Survey (CHKS), a school-based survey of 9th- and 11th-graders attending California schools. A majority (approximately 85%) of California public school districts self-select to participate, often collecting CHKS data every other school year.^21,22^ Schools are sampled for representativeness, with district-level studies reporting more than an 85% student response rate.^23,24^ With parental passive consent, all students or a randomly selected representative sample of students in grades 7, 9, and 11 are invited to complete the online survey in class. Surveys take typically 15 to 30 minutes to complete and are offered in English or Spanish. The analytic sample for this study included 9th- and 11th-grade students at 4-year public high schools (excluding 4.9%) who provided a valid answer (ie, yes, no) on the suicide outcome (excluding 6.5%; n = 557 085). We included adolescents who identified as boy or girl (excluding 1.7%) for gender-specific analysis or regressions adjusting for gender (n = 547 816).

### Measures

The CHKS includes 3 self-reported race and ethnicity questions: (1) Hispanic or Latino origin; (2) 5 mutually exclusive racial and 1 multiracial category, and (3) 11 Asian and Native Hawaiian or Pacific Islander categories with a mark-all-that-apply option (eAppendix in Supplement 1). We combined questions 1 and 2 to create ethnoracialized groups of Hispanic or Latinx, non-Hispanic American Indian or Alaska Native, non-Hispanic Black, non-Hispanic White, and multiracial (unspecified). For Asian and Native Hawaiian or Pacific Islander groups, adolescents who selected both Asian question 2 and Asian ethnicities question 3 were categorized into Asian Indian, Chinese, Filipino, Japanese, Korean, Southeast Asian (specifically, Cambodian, Hmong, or Laotian), Vietnamese, and other Asian group (unspecified). While Vietnamese is considered part of the Southeast Asian group, this group was treated as a separate group given that these individuals represent the fourth largest Asian group in California and have a large sample size in CHKS. Adolescents who selected Native Hawaiian or Pacific Islander in question 2 or Native Hawaiian or Pacific Islander in question 3 (including Native Hawaiian, Guamanian, Samoan, Tahitian, or other Pacific Islander) were categorized into the Native Hawaiian or Pacific Islander group.

With question 3’s mark-all-that-apply option, we further distinguished participants who selected a single Asian or Native Hawaiian or Pacific Islander identification and those who selected multiple Asian or Native Hawaiian or Pacific Islander identifications (eAppendix in Supplement 1). Categories for each Asian subgroup included monoethnic or monoracial Asian, multiethnic Asian, and multiracial Asian (including American Indian or Alaska Native, Black, Hispanic or Latinx, White, and multiracial [unspecified] but not Native Hawaiian or Pacific Islander). For Native Hawaiian or Pacific Islander adolescents, categories included monoracial Native Hawaiian or Pacific Islander, multiracial Asian and Native Hawaiian or Pacific Islander, and multiracial Native Hawaiian or Pacific Islander (including American Indian or Alaska Native, Black, Hispanic or Latinx, White, and multiracial but not Asian).

The primary outcome was suicidal ideation captured by the question, “During the past 12 months, did you ever seriously consider attempting suicide?” Adolescent-level covariates were sex (female in reference to male), grade (11th in reference to 9th), and parental education (high school degree, some college, college degree or higher, and unknown [representing 13.5% of the total sample] in reference to less than high school).

### Statistical Analysis

The data analysis was performed between January 5, 2023, and March 31, 2024. A series of descriptive (with observed prevalence and 95% CIs) and logistic regression analyses were conducted. First, we followed a traditional approach by examining differences in suicidal ideation prevalence between all ethnoracialized groups and an aggregated Asian and Native Hawaiian or Pacific Islander group. Second, to investigate heterogeneity within and between groups, we implemented the following disaggregation approaches: (1) separating Asian and Native Hawaiian or Pacific Islander into 2 distinct racialized groups and (2) further separating Asian into ethnic subgroups, including Asian Indian, Chinese, Filipino, Japanese, Korean, Southeast Asian, Vietnamese, and other Asian. We also distinguished between monoethnic Asian, multiethnic Asian, and multiracial Asian (except for Native Hawaiian or Pacific Islander) and separately for Native Hawaiian or Pacific Islander, distinguishing monoracial, multiracial Asian and Native Hawaiian or Pacific Islander, and multiracial Native Hawaiian or Pacific Islander (except for Asian).

#### Between-Group Differences for All Ethnoracialized Groups

Logistic regression models were used to test for differences of suicidal ideation among ethnoracialized groups, adjusting for covariates. In the traditional approach, mutually exclusive ethnoracialized groups of American Indian or Alaska Native, aggregated Asian and Native Hawaiian or Pacific Islander, Black, Hispanic or Latinx, and multiracial were compared using White adolescents as the reference group in model 1. In the disaggregated approach, models 2 and 3 separated Asian and Native Hawaiian or Pacific Islander adolescents and further by monoethnic, multiethnic, and multiracial identifications.

#### Within- and Between-Group Differences for Asian and Native Hawaiian or Pacific Islander Groups

Logistic regression models were fit to compare suicidal ideation within Asian and between Asian and Native Hawaiian or Pacific Islander groups. Given a large multiethnic Asian group, 2 different regression approaches were performed. Model 1 applied a standard approach of ethnoracialized comparisons by entering mutually exclusive Asian dummy variables (representing monoethnic Asian or Native Hawaiian or Pacific Islander) and a separate general multiethnic Asian dummy variable and multiracial Asian and Native Hawaiian or Pacific Islander dummy variable. Models used Chinese adolescents (one of the largest groups and with a lower prevalence of suicidal ideation) as the referent.

Our second approach explored an additive model allowing multiple ethnoracialized endorsements by entering nonmutually exclusive Asian ethnicity dummy variables. For example, an adolescent who is monoethnic Chinese is coded 1 for Chinese and 0 for all other ethnicities. In contrast, an adolescent who is multiethnic Chinese and Filipino is coded 1 for each identity of Chinese, Filipino, and multiethnic Asian, and 0 for all other ethnicities. The mutually exclusive model 1 and additive model 2 were compared in terms of model fit following Klein et al^25^ and by the observed and estimated prevalence of suicidal ideation across Asian subgroups (eAppendix in Supplement 1).

All analyses were conducted using Stata, version 17 (StataCorp LLC).^26^ Statistical significance was considered reached at *P* < .05. To adjust for potential correlation among students from clustering within schools, all CIs for estimates of suicidal ideation prevalence were generated using Stata survey command, treating schools as primary sampling units, and odds ratios (ORs) with 95% CIs in logistic regressions were based on robust standard error estimation, treating schools as clusters.

## Results

Pooled across 2017-2018 and 2018-2019 CHKS data, the overall analytic sample included 557 085 9th- or 11th-grade students (49.7% boys and 50.3% girls) from 791 high schools, of whom 77 735 (13.9%) identified as Asian or Native Hawaiian or Pacific Islander (compared with 3.6% identifying as American Indian or Alaska Native, 4.0% as Black, 46.3% as Hispanic or Latinx, 22.8% as White, and 9.4% as multiracial [unspecified]). Participant characteristics are presented in eTable 1 in Supplement 1.

In the disaggregation process, a large proportion of Asian and Native Hawaiian or Pacific Islander adolescents endorsed multiethnic Asian (13.8%) or multiracial (38.3%) identities. Japanese adolescents (n = 9710) had the highest proportion of multiethnic (1533 [15.8%]) and multiracial (6733 [69.3%]) Asian identities. In contrast, Asian Indian adolescents had the highest proportion of monoethnic identity (9896 of 13 355 [74.1%]), followed by Vietnamese (10 327 of 16 776 [61.6%]) and Korean (5115 of 9212 [55.5%]) adolescents. Among 11 622 Native Hawaiian or Pacific Islander adolescents, 3608 (31.0%) identified as monoracial, 1360 (11.7%) as both Asian and Native Hawaiian or Pacific Islander, and 6654 (57.3%) as multiracial Native Hawaiian or Pacific Islander.

### Suicidal Ideation Risk

#### Between-Group Differences for All Ethnoracialized Groups

**Figure 1.**
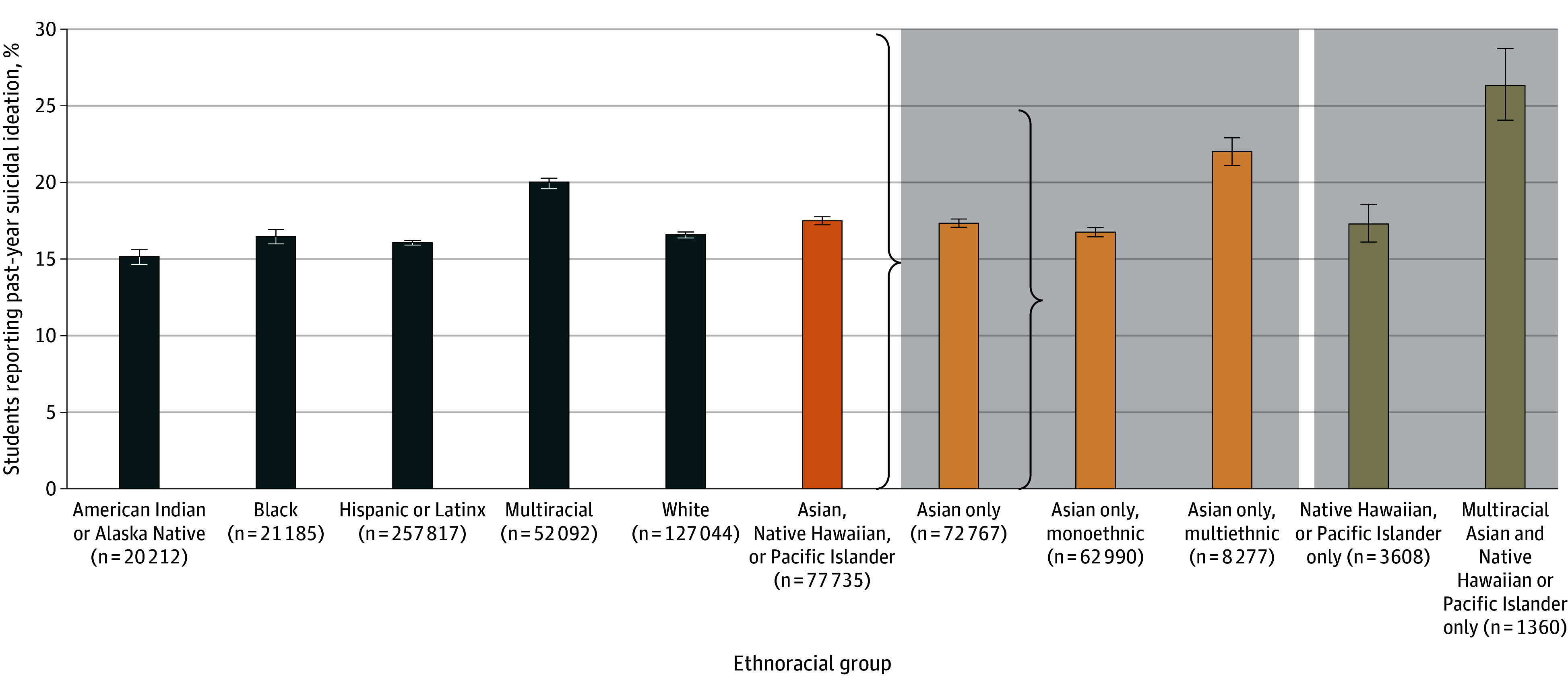
Prevalence of Suicidal Ideation by Ethnoracialized Groups Further Disaggregated by Asian American, Native Hawaiian, and Pacific Islander Groups, 2017-2018 and 2018-2019 California Healthy Kids Survey (N = 557 085) Multiracial Asian and Native Hawaiian or Pacific Islander includes students who identified as both Asian and Native Hawaiian or Pacific Islander but did not identify with any other ethnoracialized group. Numbers for monoethnic Asian, multiethnic Asian, Native Hawaiian or Pacific Islander, and multiracial Asian and Native Hawaiian or Pacific Islander do not add up to the combined Asian American, Native Hawaiian, and Pacific Islander population due to missing values. Whiskers indicate the 95% CI.

Figure 1 presents the prevalence of past-year suicidal ideation by ethnoracialized groups (N = 547 816 students). Prevalence of suicidal ideation in the combined Asian and Native Hawaiian or Pacific Islander group (13 599 of 77 735) was 17.5% (95% CI, 17.1%-17.9%), higher than all other ethnoracialized groups except the multiracial group (10 386 of 52 092 [19.9%; 95% CI, 19.5%-20.4%]). In model 1 (Table 1), results confirmed a higher odds of suicidal ideation (OR, 1.08; 95% CI, 1.04-1.12) for Asian and Native Hawaiian or Pacific Islander adolescents combined compared with White adolescents.

**Table 1.** Logistic Regression Models of Suicidal Ideation Among California Adolescents (n = 547 816)^a^

Characteristic	Model, OR (95% CI)		
	1	2	3
Asian and Native Hawaiian or Pacific Islander combined	1.08 (1.04-1.12)	NA	NA
Asian only	NA	1.07 (1.03-1.11)	NA
Monoethnic	NA	NA	1.03 (0.99-1.07)
Multiethnic	NA	NA	1.41 (1.33-1.50)
Native Hawaiian or Pacific Islander only	NA	1.05 (0.96-1.15)	1.05 (0.96-1.15)
Native Hawaiian or Pacific Islander and Asian, multiracial	NA	1.77 (1.57-2.00)	1.77 (1.57-2.00)
American Indian or Alaska Native	0.88 (0.80-0.97)	0.88 (0.80-0.97)	0.88 (0.80-0.97)
Black	1.00 (0.95-1.05)	1.00 (0.95-1.05)	1.00 (0.95-1.05)
Hispanic or Latinx	0.92 (0.89-0.95)	0.92 (0.89-0.95)	0.92 (0.89-0.95)
White	1 [Reference]	1 [Reference]	1 [Reference]
Multiracial (unspecified)	1.27 (1.23-1.31)	1.27 (1.23-1.31)	1.27 (1.23-1.31)

Abbreviations: NA, not applicable as this characteristic was not included in the regression model; OR, odds ratio.

^a^ Pooled data from the 2017-2018 and 2018-2019 California Healthy Kids Survey. All models include other racial and ethnic groups (American Indian or Alaska Native, Black, Hispanic or Latinx, or multiracial [unspecified]) with White as the reference group, adjusting for sex, school grade, and parental education.

When Asian adolescents were further divided into monoethnic and multiethnic identifications, 22.0% (1822 of 8277; 95% CI, 21.1%-23.0%) of multiethnic Asian adolescents reported suicidal ideation compared with 16.8% (10 554 of 62 990; 95% CI, 16.3%-17.2%) of monoethnic Asian adolescents (eTable 2 in Supplement 1). A similar pattern was observed for Native Hawaiian or Pacific Islander adolescents, among whom those who identified as both Native Hawaiian or Pacific Islander and Asian had a higher prevalence of suicidal ideation (358 of 1360 [26.3%; 95% CI, 24.1%-28.7%]) compared with those who identified as monoracial Native Hawaiian or Pacific Islander (624 of 3608 [17.3%; 95% CI, 16.1%-18.6%]). As shown in Table 1, both multiethnic Asian and multiracial Native Hawaiian or Pacific Islander and Asian adolescents had higher ORs (1.41 [95% CI, 1.33-1.50] and 1.77 [95% CI, 1.57-2.00], respectively) compared with White adolescents, while no statistically significant differences emerged for monoethnic Asian and monoracial Native Hawaiian or Pacific Islander adolescents (models 2 and 3).

#### Within- and Between-Group Differences for Asian and Native Hawaiian or Pacific Islander Groups

**Figure 2.**
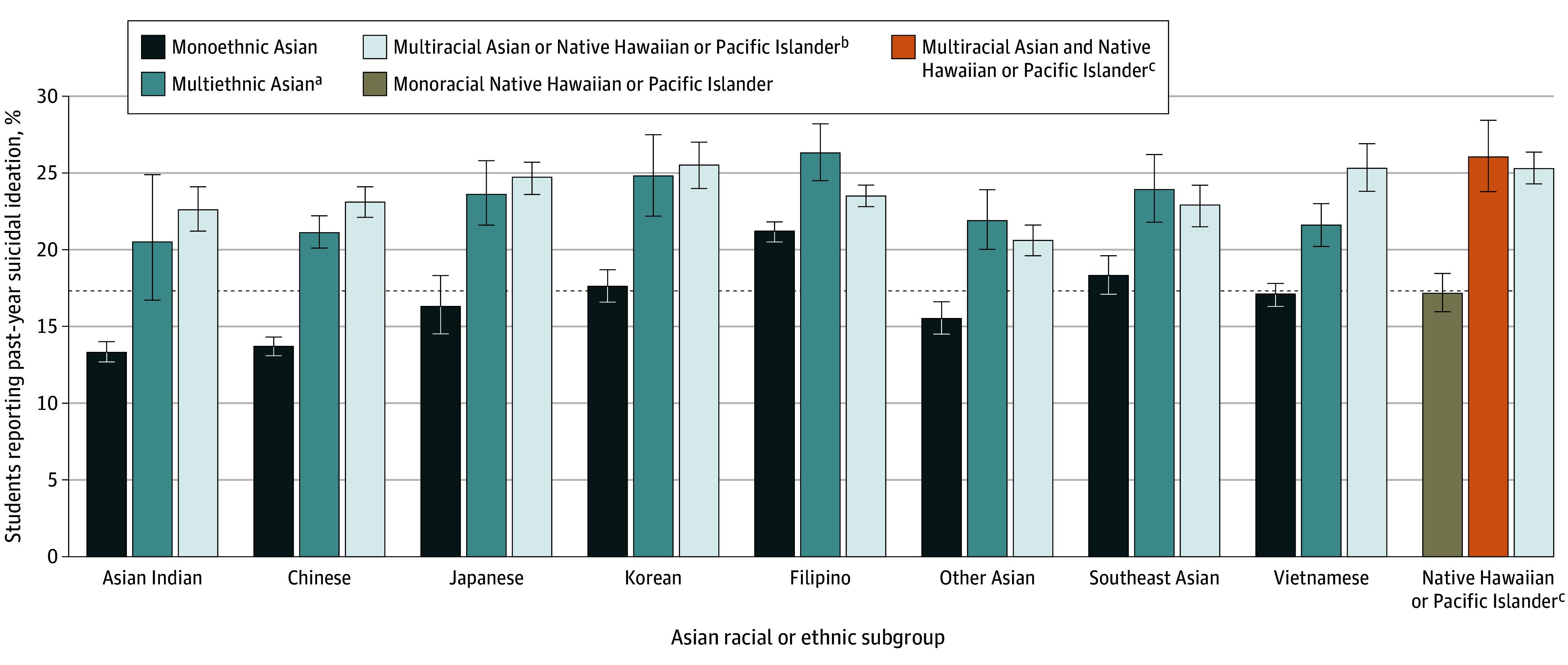
Prevalence of Suicidal Ideation by Asian, Native Hawaiian, and Pacific Islander Adolescents Disaggregated by Monoethnic, Multiethnic, and Multiracial Identification, 2017-2018 and 2018-2019 California Healthy Kids Survey (N = 77 735) Whiskers indicate the 95% CI, and the dashed line indicates the percentage of Asian and Native Hawaiian or Pacific Islander combined (17.5%; 95% CI, 17.1%-17.9%). ^a^Multiethnic Asian indicates identification with more than 1 Asian ethnic subgroup. This category is not mutually exclusive; thus, a Chinese and Southeast Asian adolescent would be included twice under Chinese and Southeast Asian. See additional notes under Native Hawaiian or Pacific Islander. ^b^Multiracial Asian or Native Hawaiian or Pacific Islander indicates identification as Asian or Native Hawaiian or Pacific Islander (but not both) and another racial and ethnic group (eg, American Indian or Alaska Native, Black, Hispanic or Latinx, White, or multiracial [unspecified]). ^c^Native Hawaiian or Pacific Islander categories are as follows: monoracial Native Hawaiian or Pacific Islander, which represents Native Hawaiian or Pacific Islander only, and multiracial Asian and Native Hawaiian or Pacific Islander, which includes adolescents who identified as both Asian and Native Hawaiian or Pacific Islander.

Figure 2 presents stratified analyses of each Asian subgroup and the Native Hawaiian or Pacific Islander group and further by monoethnic, multiethnic, and multiracial identifications. Filipino and Native Hawaiian or Pacific Islander adolescents reported the highest prevalence of suicidal ideation compared with Asian Indian and Chinese adolescents. Consistent across groups, multiethnic Asian and multiracial Asian and Native Hawaiian or Pacific Islander adolescents had higher rates of suicidal ideation compared with monoethnic Asian or monoracial Native Hawaiian or Pacific Islander adolescents, from 2.3 percentage points (from 21.2% [95% CI, 20.4%-21.9%] among monoethnic Filipino adolescents to 23.5% [95% CI, 22.8%-24.2%] among multiracial Filipino adolescents) to 9.4 percentage points (from 13.7% [95% CI, 13.0%-14.4%] among monoethnic Chinese adolescents to 23.1% [95% CI, 22.1%-24.1%] among multiracial Chinese adolescents) (eTable 3 in Supplement 1).

Table 2 compares adjusted suicidal ideation estimates from 2 mutually exclusive and 1 additive model. In the mutually exclusive models, each participant belongs to 1 Asian subgroup or Native Hawaiian or Pacific Islander group (model 1a), and if a participant is in more than 1 group, then they are also in a separate, general multiethnic Asian or multiracial Asian and Native Hawaiian or Pacific Islander group (model 1b). In the additive model (model 2), groups are nonmutually exclusive, and participants can belong to more than 1 Asian or Native Hawaiian or Pacific Islander group in addition to a general multiethnic or multiracial group. Model 1a shows that multiracial Asian and Native Hawaiian or Pacific Islander adolescents had the highest adjusted OR (2.20; 95% CI, 1.94-2.51) followed by multiethnic Asian adolescents (OR, 1.76; 95% CI, 1.63-1.90) and monoethnic Filipino adolescents (OR, 1.70; 95% CI, 1.59-1.83) compared with monoethnic Chinese adolescents as the reference group. All other monoethnic Asian subgroups had a greater odds of suicidal ideation than Chinese adolescents except for the monoethnic Asian Indian group, which was not significantly different. In model 1b, since Chinese made up a large proportion of the multiethnic Asian group, we distinguished between multiethnic Asian adolescents who selected Chinese as 1 of their multiple identifications compared with those who selected all other Asian subgroups. Non-Chinese multiethnic Asian adolescents had higher odds of suicidal ideation (OR, 2.05; 95% CI, 1.83-2.29) than Chinese multiethnic Asian adolescents (OR, 1.67; 95% CI, 1.54-1.81).

**Table 2.** Logistic Regression Model of Suicidal Ideation Among 9th- and 11th-Grade Asian and Native Hawaiian or Pacific Islander Students by Monoethnic Asian Subgroups Compared With Multiethnic Asian, Native Hawaiian or Pacific Islander, and Multiracial Asian and Native Hawaiian or Pacific Islander Groups^a^

Characteristic	Model, OR (95% CI)^b^		Model 2, OR (95% CI)^c^
	1a	1b	
Asian Indian	0.98 (0.89-1.08)	0.98 (0.89-1.08)	0.98 (0.90-1.07)
Chinese	1 [Reference]	1 [Reference]	1 [Reference]
Filipino	1.70 (1.59-1.83)	1.70 (1.59-1.83)	1.70 (1.59-1.81)
Japanese	1.25 (1.09-1.45)	1.25 (1.09-1.45)	1.27 (1.15-1.40)
Korean	1.38 (1.26-1.51)	1.38 (1.26-1.51)	1.38 (1.28-1.49)
Other Asian	1.16 (1.04-1.29)	1.16 (1.04-1.29)	1.19 (1.10-1.29)
Southeast Asian	1.37 (1.22-1.55)	1.37 (1.22-1.55)	1.34 (1.22-1.48)
Vietnamese	1.28 (1.16-1.40)	1.28 (1.16-1.40)	1.27 (1.18-1.38)
Multiethnic Asian	1.76 (1.63-1.90)	NA	1.16 (1.08-1.23)
Chinese	NA	1.67 (1.54-1.81)	NA
Not Chinese	NA	2.05 (1.83-2.29)	NA
Native Hawaiian or Pacific Islander only	1.29 (1.16-1.43)	1.29 (1.16-1.43)	1.28 (1.16-1.42)
Multiracial Asian and Native Hawaiian or Pacific Islander	2.20 (1.94-2.51)	2.20 (1.94-2.51)	2.20 (1.94-2.50)
Model fit			
No., *df*	75 360, 17	75 360, 18	75 360, 17
Log likelihood	−34 161.25	−34 155.72	−34 130.00
AIC	68 356.51	68 347.45	68 293.99
BIC	68 513.42	68 513.59	68 450.90

Abbreviations: AIC, Akaike information criterion; BIC, bayesian information criterion; NA, not applicable as this characteristic was not included in the regression model; OR, odds ratio.

^a^ Pooled data from the 2017-2018 and 2018-2019 California Healthy Kids Survey. All models control for sex, school grade, and parental education.

^b^ Models 1a and 1b have Asian subgroup indicators coded as mutually exclusive dummy variables (eg, Chinese means Chinese only), and Asian adolescents who reported more than 1 group (eg, Chinese and Filipino) belong to multiethnic Asian.

^c^ Model 2 is an additive model with the dichotomous Asian subgroup indicators and not mutually exclusive. For example, an adolescent who is Chinese only is coded 1 for Chinese and 0 for all other indicators. In contrast, an adolescent reporting Chinese and Filipino is coded 1 for Chinese, Filipino, and multiethnic Asian and 0 for all other indicators. Note that Native Hawaiian or Pacific Islander only and multiracial Asian and Native Hawaiian or Pacific Islander are still coded as mutually exclusive indicators (between them and with Asian subgroup indicators) as in models 1 and 2.

Model 2 estimates were similar to model 1 except for the multiethnic Asian group, which represented an added effect rather than an independent effect as in model 1a. As an example, we have 2 multiethnic Asian adolescent groups, 1 being Chinese and Filipino and the other Filipino and Vietnamese. Based on model 1a, both adolescent groups were estimated to have 76% higher odds of suicidal ideation compared with monoethnic Chinese adolescents. Based on model 1b, Chinese and Filipino adolescents had 67% higher odds and Filipino and Vietnamese adolescents had 105% higher odds than monoethnic Chinese adolescents. Model 2 fits an additive model, which allowed us to derive effect estimates for multiple identities simply by multiplying their ORs. In model 2, Chinese and Filipino adolescents had 97% higher odds of suicidal ideation (OR, 1.70 × 1.16, being Filipino and multiethnic Asian), while Filipino and Vietnamese adolescents had a 150% higher odds (OR, 1.70 × 1.27 × 1.16, being Filipino, Vietnamese, and multiethnic Asian) compared with monoethnic Chinese adolescents.

Based on model fit indexes and comparisons of the observed prevalence of suicidal ideation and the model-estimated marginal prevalence (Table 2; eTable 4 in Supplement 1), model 2 showed the best fit. Mutually exclusive models 1a and 1b for monoethnic Asian adolescents perfectly estimated the observed value, and minimal differences were found between observed and estimated values in the additive model (model 2 in eTable 4 in Supplement 1). These smaller absolute differences showed that the estimative performance of model 2 was much better than models 1a and 1b for multiethnic Asian subgroups, suggesting that an additive model is favorable.

## Discussion

Although suicide is one of the leading causes of death for Asian, Native Hawaiian, and Pacific Islander adolescents,^1^ suicidal behaviors among these adolescents have been overlooked as a pressing public health problem. This lack of visibility may be partially due to systematic biases in data collection and availability wherein Asian and Native Hawaiian or Pacific Islander groups tend to be aggregated, concealing the heterogeneity of risks. This cross-sectional study addressed these inequities by investigating risk of suicidal ideation within and between Asian and Native Hawaiian or Pacific Islander adolescents through data disaggregation.

The study findings can be summarized into 3 key points. First, data disaggregation revealed significant differences between Asian and Native Hawaiian or Pacific Islander adolescents. Adolescents who identified as Filipino, Korean, Southeast Asian, and Native Hawaiian or Pacific Islander had significantly higher odds of suicidal ideation compared with Chinese and Asian Indian adolescents. Second, we further distinguished Asian and Native Hawaiian or Pacific Islander adolescents into monoethnic, multiethnic, and multiracial subgroups. When using a traditional between-ethnoracialized group analysis, an aggregated Asian and Native Hawaiian or Pacific Islander group showed significantly higher odds of suicidal ideation than White adolescents. Upon examination of within-group differences, adolescents who identified with more than 1 Asian subgroup or as both Asian and Native Hawaiian or Pacific Islander consistently had a much higher suicidal ideation rate than adolescents who identified as monoethnic or monoracial, regardless of which Asian subgroup or Native Hawaiian or Pacific Islander group. Third, in using these rich data with large sample sizes, the process of data disaggregation was complex and required important decisions around grouping the ethnoracialized categorizations and analytic approaches, which have important implications for study results.

Adolescents’ self-reported identification with a single or multiple ethnoracialized categories represents a proxy for their social and cultural experiences as it relates to their ethnic identity. We found that Filipino, Korean, and Southeast Asian adolescents have the highest risk of suicidal ideation followed by Native Hawaiian or Pacific Islander, Vietnamese, and Japanese adolescents. Identifying as Asian or Native Hawaiian or Pacific Islander may elevate risks of suicidal ideation due to stress or social discord experienced by ethnoracialized groups,^27^ yet how these play out for each Asian subgroup and Native Hawaiian or Pacific Islander adolescents is unknown. Future research is needed to consider how experiences of historical trauma (US colonization for Native Hawaiian or Pacific Islander and Filipino people), being a refugee (Southeast Asian and Vietnamese people), and US internment (Japanese people) may have affected younger generations,^28^ and despite seemingly successful settlements, how interpersonal and structural racism (ie, Muslim ban, anti-Asian hate crimes) have contributed to Asian and Native Hawaiian or Pacific Islander adolescent mental health.^29,30^ Ethnic subgroup identity may moderate culturally specific experiences, race-related stressors, or social exclusion that heighten risk for suicidal behaviors.

Importantly, across all groups, including those that exhibited a lower prevalence of suicidal ideation, Asian and Native Hawaiian or Pacific Islander adolescents with multiple identifications exhibited the highest risk of suicidal ideation. This finding is not new. A growing number of studies has reported that multiracial adolescents have poorer mental health and more suicidal thoughts and behaviors than their monoracial counterparts.^7,31^ Our findings show that this elevated risk of suicidal ideation is not limited to Asian or Native Hawaiian or Pacific Islander adolescents who also identified as American Indian or Alaska Native, Black, Hispanic or Latinx, or White but extends to Asian or Native Hawaiian or Pacific Islander adolescents who identified with more than 1 Asian subgroup or as multiracial Asian and Native Hawaiian or Pacific Islander. Belonging to multiple ethnoracialized groups, even when groups fall under the Asian umbrella term, may highlight within-group discord and contribute to adolescents’ sense of belonging, particularly in environments where social groups are defined prominently through ethnoracialized lenses. This possibility of a conflicted identity and lack of belonging in multiple ethnoracialized groups are in line with the interpersonal theory of suicide, which points to thwarted belongingness and social isolation as critical suicidal precipitants.^32,33^ Given changing youth demographics with multiracial identities,^16^ how Asian and Native Hawaiian or Pacific Islander adolescents define belonging (both for monoethnic and multiethnic and multiracial) and, in particular, the groups or communities to which they feel they belong will be important for future research toward developing suicide prevention and intervention.

Our final study conclusion calls for data equity by collecting, analyzing, and disseminating data that best reflect population diversity.^34^ We recommend a public health critical race praxis in the construction of ethnoracialized groups and statistical approaches.^35^ In pooling 2 years of CHKS data from a large, diverse population of California adolescents by asking specific questions on Asian subgroup and Native Hawaiian or Pacific Islander identity, we had a valuable opportunity for deeper investigation. In addition to separating Asian and Native Hawaiian or Pacific Islander as 2 distinct racialized groups (regardless of sample size), we identified further multiethnic and multiracial identities and captured a substantial number of Filipino adolescents who reported their racialized identification as Native Hawaiian or Pacific Islander rather than Asian (despite US Office of Management and Budget designations^3^), which may all be easily overlooked when data are unavailable or inaccessible (see eAppendix in Supplement 1). Finally, to advance analytic opportunities, we present a couple approaches: (1) a comparison of between-group estimates when Asian and Native Hawaiian or Pacific Islander groups are aggregated and disaggregated and (2) a comparison of a mutually exclusive approach with a single multiethnic category vs an additive approach that accounts for multiple identities. Through model fit and comparisons of observed and estimated values, the additive model best captured the data. By continuing to use a mutually exclusive approach or creating a single multiethnic or multiracial group, the complexities of one’s identity could remain hidden.

### Limitations

Future research should expand beyond suicide ideation to consider other suicidal behaviors (eg, plans, attempts) and consider psychological and contextual factors that may elevate risks of suicidal ideation. Racial and ethnic identity were self-reported and subject to bias given that youth are still exploring and developing their ethnic identity. Thus, demographic characteristics, such as generational status or languages spoken, and sense of cultural belonging are important for data validity. Although disaggregation was primarily focused on Asian subgroups, Cambodian, Hmong, and Laotian adolescents were aggregated into 1 Southeast Asian group for sample size; further studies should examine them separately. In addition, data were lacking to disaggregate Native Hawaiian or Pacific Islander subgroups. The CHKS data may not be representative of adolescents attending public high schools in California given that schools may not have equal probabilities in electing to participate. Finally, we acknowledge our own research biases situated within a traditional disparities lens through which the dominant or majority groups serve as the normative reference and statistical approaches often pit groups against each other for comparison. We attempted to showcase different approaches to reduce these biases.

## Conclusions

To our knowledge, this cross-sectional study is the first to systematically examine suicidal ideation across Asian Indian, Chinese, Filipino, Japanese, Korean, Southeast Asian, Vietnamese, and Native Hawaiian or Pacific Islander adolescents and further by monoethnic, monoracial, multiethnic, and multiracial identifications. Too often, Asian and Native Hawaiian or Pacific Islander adolescents are overlooked, but as our findings show, Asian and Native Hawaiian or Pacific Islander adolescent suicidal ideation is a serious public health issue that must be addressed through adequate mental health research, intervention, and health services funding. To develop population-level strategies and policies, collection and reporting of disaggregated ethnoracialized data are crucial to identify, prioritize, and understand experiences of individuals at risk for suicide. Future suicide research and prevention and intervention strategies should deepen investigations of belonging and ethnic identity, in particular multiple identifications, and uncover how these experiences may be leveraged to reduce suicidal ideation among Asian and Native Hawaiian or Pacific Islander adolescents.

## Notes

Supplement 1. **eAppendix.** Race and Ethnicity Question in California Healthy Kids Survey (CHKS), 2017/18 and 2018/19, and Classification of Asian and Native Hawaiian or Pacific Islander Groups **eTable 1.** Demographic Characteristics of California Healthy Kids Survey Participants **eTable 2.** Prevalence of Suicidal Ideation by Ethnoracialized Groups, Complementary to Figure 1 **eTable 3.** Prevalence of Suicidal Ideation by Asian and Native Hawaiian or Pacific Islander Identification, Complementary to Figure 2 **eTable 4.** Observed and Predicted Suicidal Ideation Prevalence for Asian Subgroups Across Models in Table 2

Supplement 2.
Data Sharing Statement
