# Supplementary material for: Unmasking Suicidal Ideation for Asian American, Native Hawaiian, and Pacific Islander Youths Via Data Disaggregation
Source: JAMA Netw Open. 2024 Nov 22;7(11):e2446832. doi: 10.1001/jamanetworkopen.2024.46832 (PMC11584931; doi:10.1001/jamanetworkopen.2024.46832)
Supplement: Supplement 1. — eAppendix. Race and Ethnicity Question in California Healthy Kids Survey (CHKS), 2017/18 and 2018/19, and Classification of Asian and Native Hawaiian or Pacific Islander Groups eTable 1. Demographic Characteristics of California Healthy Kids Survey Participants eTable 2. Prevalence of Suicidal Ideation by Ethnoracialized Groups, Complementary to Figure 1 eTable 3. Prevalence of Suicidal Ideation by Asian and Native Hawaiian or Pacific Islander Identification, Complementary to Figure 2 eTable 4. Observed and Predicted Suicidal Ideation Prevalence for Asian Subgroups Across Models in Table 2 [file jamanetwopen-e2446832-s001.pdf]

Supplemental Online Content

Lui CK, Ye Y, Gee J, et al. Unmasking suicidal ideation for Asian, Native Hawaiian, and Pacific Islander youths via data diaggregation. *JAMA Netw Open*. 2024;7(11):e2446832. doi:10.1001/jamanetworkopen.2024.46832

eAppendix. Race and Ethnicity Question in California Healthy Kids Survey (CHKS), 2017/18 and 2018/19, and Classification of Asian and Native Hawaiian or Pacific Islander Groups

eTable 1. Demographic Characteristics of California Healthy Kids Survey Participants

eTable 2. Prevalence of Suicidal Ideation by Ethnoracialized Groups, Complementary to Figure 1

eTable 3. Prevalence of Suicidal Ideation by Asian and Native Hawaiian or Pacific Islander Identification, Complementary to Figure 2

eTable 4. Observed and Predicted Suicidal Ideation Prevalence for Asian Subgroups Across Models in Table 2

This supplemental material has been provided by the authors to give readers additional information about their work.

© 2024 Lui CK et al. *JAMA Network Open.*

eAppendix. Race and Ethnicity Questions in California Healthy Kids Survey, 2017/18 and 2018/19, and classification of Asian and Native Hawaiian or Pacific Islander groups

California Healthy Kids Survey Questions

1. Are you of Hispanic or Latino origin?
   - A) No
   - B) Yes

2. What is your race? ***Select only one***
   - A) American Indian or Alaska Native
   - B) Asian
   - C) Black or African American
   - D) Native Hawaiian or Pacific Islander
   - E) White
   - F) Mixed (two or more) races

3. If you are Asian or Pacific Islander, which groups best describe you? ***Mark all that apply***
   If you are **<u>not</u>** of Asian/Pacific Islander background, mark "A) Does not apply."
   - A) Does not apply; I am not Asian or Pacific Islander
   - B) Asian Indian
   - C) Cambodian
   - D) Chinese
   - E) Filipino
   - F) Hmong
   - G) Japanese
   - H) Korean
   - I) Laotian
   - J) Vietnamese
   - K) Native Hawaiian, Guamanian, Samoan, Tahitian, or other Pacific Islander
   - L) Other Asian

Methods on Data Disaggregation:

We distinguished between a single Asian or Native Hawaiian and Pacific Islander identification and multiple Asian or Native Hawaiian or Pacific Islander identification: **<u>Single or monoethnic/monoracial Asian</u>** group included those who selected Asian in Q2 and identified as only one Asian subgroup in Q3 : **Asian Indian, Chinese, Filipino, Japanese, Korean, other Asian, Southeast Asian (including Cambodian, Hmong, Laotian), and Vietnamese**. The **<u>multiethnic Asian</u>** group included those who selected as Asian in Q2, and selected more than one Asian subgroup in Q3. Note that among those who reported Asian race (Q2), a small number (2.3%) did not check any of the Asian subgroups (Q3). Finally, **<u>multiracial Asian</u>** accounted for multiracial identities, or those who selected one or more Asian subgroups in Q3 and also identified as Hispanic or Latinx (Q1) or as American Indian or Alaska Native, Black or African American, multiracial, or White (Q2). For Native Hawaiian or Pacific Islander adolescents, a similar process occurred. **<u>Single or monoracial Native Hawaiian or Pacific Islander</u>** adolescents included those who identified as Native Hawaiian or Pacific Islander in Q2 and selected no

© 2024 Lui CK et al. *JAMA Network Open.*

Asian group in Q3. We further divided multiracial Native Hawaiian or Pacific Islander into **multiracial Native Hawaiian or Pacific Islander and Asian** (those who only identified as Asian or Native Hawaiian or Pacific Islander in Q2 and selected Asian subgroup or Native Hawaiian or Pacific Islander in Q3) and **multiracial Native Hawaiian or Pacific Islander** (those who selected Native Hawaiian or Pacific Islander in Q2 or Q3 and also identified as Hispanic or Latinx in Q1 or as American Indian or Alaska Native, Black, multiracial or White in Q2).

Special care was taken when defining Native Hawaiian or Pacific Islander and its subgroups, due to high inconsistency between Q2 and Q3 related to Native Hawaiian or Pacific Islander. Of 9603 students who were identified as Native Hawaiian or Pacific Islander in Q2, only 3369 checked Native Hawaiian or Pacific Islander in Q3. Further, of the 6234 students who reported as Native Hawaiian or Pacific Islander in Q2 but not in Q3, only 1,016 (16.3%) did not report any Asian subgroup in Q3, while the remaining 5218 students checked at least one Asian subgroup (4928 reported one Asian subgroup and 290 more than one subgroup), with the vast majorities of them (n=4518, 91.1%) checking Filipino. Therefore, despite selecting Native Hawaiian or Pacific Islander race in Q2, these 4518 adolescents were categorized as Asian and Filipino because they only selected Filipino in Q3 and the Office of Management and Budget (OMB) categorizes Filipinos under Asian race.[3]

Due to the observed high inconsistency between Q2 and Q3 on Native Hawaiian or Pacific Islander, particularly the possible miss-classification of Filipino to Native Hawaiian or Pacific Islander (e.g. see Office of Management and Budget (OMB)), we define the Native Hawaiian or Pacific Islander-related subgroups as below. **Single or monoracial Native Hawaiian or Pacific Islander** adolescents included those who identified as Native Hawaiian or Pacific Islander in both Q2 and Q3, or those who identified as Native Hawaiian or Pacific Islander in Q2 but not Q3 and did not select any Asian subgroup in Q3. **Asian and Native Hawaiian or Pacific Islander** included those who identified as Asian or Native Hawaiian or Pacific Islander in Q2, and selected Native Hawaiian or Pacific Islander in Q3 and at least one Asian subgroup in Q3. Those who identified as Native Hawaiian or Pacific Islander in Q2 but not in Q3 and selected at least one Asian subgroup (n=5218, described above) were not defined as Native

© 2024 Lui CK et al. *JAMA Network Open.*

Hawaiian or Pacific Islander, but as single or multiethnic Asians instead. Last, **multiracial Native Hawaiian or Pacific Islander** included those who selected Native Hawaiian or Pacific Islander in Q3 and selected neither Native Hawaiian or Pacific Islander nor Asian in Q2 (American Indian or Alaska Native, Black, multiracial, or White, etc.). Survey participant characteristics are presented in eTable 1.

Summary of Criteria for Monoethnic, Multiethnic and Multiracial categories

Category	Criteria
**Asian**	
**1. Single/Monoethnic Asian**<br>a. Asian Indian<br>b. Chinese<br>c. Filipino<br>d. Japanese<br>e. Korean<br>f. Other Asian (not specified)<br>g. Other Southeast Asian (e.g., Cambodian, Hmong, Laotian)<br>h. Vietnamese	Identified as Asian in Q2 **and** selected only *one* Asian subgroup in Q3
**2. Multiethnic Asian**<br>Identifies with more than one group from 1a-h.	Identified as Asian in Q2 **and** selected *more than one* Asian subgroup in Q3.
**3. Multiracial Asian**<br>Identifies with one or more group from 1a-h.	Selected one or more Asian subgroups in Q3 **and** also identified as Hispanic or Latinx (Q1) or as American Indian or Alaska Native, Black, multiracial, or White (Q2).<br><br>This group is included in the descriptive prevalence, but is not included in the regression analyses due to potential bias from the mutually exclusive categories in Q2.
**Native Hawaiian or Pacific Islander**	
**1. Single/Monoracial Native Hawaiian or Pacific Islander**	Identified as Native Hawaiian or Pacific Islander in Q2 or Q3, **and** selected no Asian group in Q3.
**2. Multiracial Asian and Native Hawaiian or Pacific Islander**	Identified as Asian or Native Hawaiian or Pacific Islander in Q2, **and** selected Native Hawaiian or Pacific Islander and at least one Asian subgroup in Q3.
**3. Multiracial Native Hawaiian or Pacific Islander** (not including Asian)	Selected Native Hawaiian or Pacific Islander in Q3 **and** identified as Hispanic or Latinx in Q1and/or American Indian or Alaska Native, Black, multiracial, or White in Q2.<br><br>This group is included in the descriptive prevalence, but is not included in the regression analyses due to potential bias from the mutually exclusive categories in Q2.

Notes: Among those who reported Asian race (Q2), a small number (2.3%) did not check any of the Asian subgroups (Q3).

© 2024 Lui CK et al. *JAMA Network Open.*

Analyses: Comparing Model fit from Table 2 using observed and predicted values:

Comparing model fit indices (Table 2), Model 1b performed slightly better than Model 1a, and Model 2 performed substantially better than Models 1a and 1b. As the two types of models have both advantages and disadvantages, we further evaluated their prediction performance, by comparing the observed suicide ideation prevalence and the model-predicted marginal prevalence, separately conducted for the monoethnic Asian subgroups and the multiethnic Asians. For the eight monoethnic Asian groups (Asian Indians to Other Asians, observed suicide ideation prevalence ranging from 13.3% to 21.1%), the mutually exclusive models (1a and 1b) perfectly predicted the marginal prevalence as expected. The additive model performed well in prediction, with the absolute difference between the observed and predicted prevalence ranging from 0.01% to 0.41%. Across the eight monoethnic groups, the simple average in difference was 0.14% and the weighted average was 0.08%. To evaluate prediction performance for the multiethnic Asians (N=8190), we sorted the sample based on combinations of subgroup endorsement and select the ten largest groups (Chinese + Vietnamese the largest (N=2214), Chinese + Filipino the second (N=1028), etc.) together with the remaining group (N=2103). The observed prevalence of suicide ideation across these eleven groups ranged from 14.5% to 28.7%. For these multiethnic Asian subgroups, the prediction performance of Model 2 was much better than Model 1a and 1b. Across groups, the simple average of absolute difference between the observed and predicted prevalence was 1.6% from Model 2, compared to 4.2% and 3.6% from Model 1a and 1b, respectively. Similarly, the weighted average was 1.1% from Model 2, compared to 3.8% and 3.0% from Model 1a and 1b (See eTable 4).

Data Limitations: By allowing students to select only one race in Question #2 can lead to undercount of specific groups like American Indian or Alaska Native where multiracial identities make up a larger proportion of their population. Since 2020-2021, the race and ethnicity questions in CHKS has been revised such that students can mark all that apply in Question #2 which will allow for a better understanding of the mixed or multiracial identities.

© 2024 Lui CK et al. *JAMA Network Open.*

eTable 1. Demographic Characteristics of California Healthy KidsS Survey Participants (n=557,085)[a]

Characteristic	Boys	9th grade	Parental education					Total n
			<HS grad	HS grad	Some college	College grad	Unknown/ Missing	
**Traditional approach**								
American Indian or Alaska Native	51.2%	54.3%	27.3%	23.2%	12.8%	18.7%	18.1%	20,212
Black or African American	54.3%	52.9%	10.8%	16.2%	17.9%	41.7%	13.5%	22,185
Hispanic or Latinx	48.0%	54.6%	24.1%	22.3%	14.4%	21.7%	17.5%	257,817
Multiracial (unspecified)	52.0%	60.9%	6.1%	13.1%	15.0%	53.7%	12.2%	52,092
Non-Hispanic White	50.4%	53.1%	3.2%	10.0%	13.6%	66.4%	6.8%	127,044
Asian + Native Hawaiian or Pacific Islander combined	50.8%	52.7%	5.8%	10.4%	9.8%	63.1%	10.9%	77,735
**Disaggregated approach #1**								
Asian only	50.7%	52.7%	5.4%	9.7%	9.4%	64.9%	10.6%	72,767
* Asian only, single identification	50.9%	52.3%	5.3%	9.6%	9.2%	65.1%	10.8%	62,990
* Asian only, multiple identification	48.2%	56.4%	5.5%	9.4%	11.2%	64.5%	9.4%	8,277
Native Hawaiian or Pacific Islander all	52.3%	53.2%	11.0%	21.6%	15.8%	37.0%	14.6%	4,968
* Native Hawaiian or Pacific Islander only, single identification	53.9%	52.6%	12.8%	24.3%	16.0%	30.8%	16.1%	3,608
* Multiracial Asian + Native Hawaiian or Pacific Islander	48.1%	54.9%	6.3%	14.5%	15.1%	53.5%	10.7%	1,360
**Disaggregated approach #2[b]**								
Asian Indian	49.3%	55.7%	2.9%	5.5%	4.6%	82.5%	4.5%	10,272
Chinese	48.9%	53.2%	5.4%	9.2%	7.0%	69.6%	8.8%	19,041
Filipino	51.6%	51.0%	3.3%	8.4%	12.9%	65.7%	9.8%	17,203
Japanese	53.3%	54.2%	3.9%	6.1%	6.4%	77.0%	6.6%	2,977
Korean	51.2%	53.1%	2.3%	4.0%	6.5%	80.7%	6.5%	6,117
Other Asian	51.4%	56.6%	6.8%	10.7%	9.9%	60.7%	11.8%	6,306
Southeast Asian	49.8%	52.8%	9.8%	18.8%	15.7%	35.0%	20.8%	5,167
-Cambodian	49.2%	55.1%	11.3%	18.8%	14.9%	39.8%	15.2%	1,602
-Hmong	50.6%	51.1%	9.2%	17.6%	15.1%	32.2%	25.9%	2,636
-Laotian	48.2%	55.2%	8.8%	22.8%	18.4%	34.7%	15.3%	1,223
Vietnamese	50.2%	52.8%	9.3%	14.3%	12.6%	47.4%	16.5%	13,768

[a] Pooled data from 2017/18 and 2018/19 California Healthy Kids Survey. Valid Ns excluding missing suicide ideation. Ns don't add up because of missing Asian identification. Survey participants were not asked to describe specific Asian group for the other Asian category.

[b] The total n for each Asian subgroups includes participants who identified as monoethnic or multiethnic Asian, or multiracial Asian+Native Hawaiian or Pacific Islander. They do not include participants who selected multiracial or mixed race in Q2.

© 2024 Lui CK et al. *JAMA Network Open.*

eTable 2. Prevalence of Suicidal Ideation by Ethnoracialized Groups, Complementary to Figure 1[a]

			95% CI		
**Characteristic**	**Suicide n**	**Suicide %**	**Low**	**High**	**Total n**
**Traditional approach**					
American Indian or Alaska Native	3,059	15.1%	14.0%	16.3%	20,212
Black or African American	3,649	16.4%	15.9%	17.0%	22,185
Hispanic or Latinx	41,425	16.1%	15.8%	16.4%	257,817
Multiracial (unspecified)	10,386	19.9%	19.5%	20.4%	52,092
Non-Hispanic White	21,044	16.6%	16.1%	17.0%	127,044
Asian +Native Hawaiian or Pacific Islander combined	13,599	17.5%	17.1%	17.9%	77,735
**Disaggregated approach #1**					
Asian only	12,617	17.3%	16.9%	17.8%	72,767
* Asian only, single identification	10,554	16.8%	16.3%	17.2%	62,990
* Asian only, multiple identification	1,822	22.0%	21.1%	23.0%	8,277
Native Hawaiian or Pacific Islander all	982	19.8%	18.7%	20.9%	4,968
* Native Hawaiian or Pacific Islander only, single identification	624	17.3%	16.1%	18.6%	3,608
* Multiracial Asian + Native Hawaiian or Pacific Islander	358	26.3%	24.1%	28.7%	1,360
**Disaggregated approach #2** [b]					
Asian Indian	1,396	13.6%	12.6%	14.6%	10,272
Chinese	3,068	16.1%	15.5%	16.7%	19,041
Filipino	3,750	21.8%	21.1%	22.5%	17,203
Japanese	598	20.1%	18.7%	21.5%	2,977
Korean	1,150	18.8%	17.9%	19.8%	6,117
Other Asian	1,087	17.2%	16.3%	18.2%	6,306
Southeast Asian	1,026	19.9%	18.5%	21.3%	5,167
-Cambodian	348	21.7%	19.6%	24.0%	1,602
-Hmong	491	18.6%	16.8%	20.6%	2,636
-Laotian	253	20.7%	18.4%	23.1%	1,223
Vietnamese	2,505	18.2%	17.3%	19.2%	13,768

[a] Pooled data from 2017/18 and 2018/19 California Healthy Kids Survey. Valid Ns excluding missing suicide ideation. Ns don't add up because of missing Asian identification. Survey participants were not asked to describe specific Asian group for the other Asian category.

[b] The total n for each Asian subgroup includes participants who identified as monoethnic or multiethnic Asian, or multiracial Asian+Native Hawaiian or Pacific Islander. They do not include participants who selected multiracial race in Q2.

© 2024 Lui CK et al. *JAMA Network Open.*

eTable 3. Prevalence of Suicidal Ideation by Asian and Native Hawaiian or Pacific Islander Identification, Complementary to Figure 2 [a]

	Monoethnic Asian				Multiethnic Asian[b]				Multiracial Asian[c]			
	**Suicide n**	**Mean**	95% CI	Total n	**Suicide n**	**Mean**	95% CI	Total n	**Suicide n**	**Mean**	95% CI	Total n
**Asian Indian**	**1,319**	**13.3%**	12.3%-14.4%	9,896	**77**	**20.5%**	16.7%-24.9%	376	**697**	**22.6%**	21.0%-24.3%	3,083
**Chinese**	**1,756**	**13.7%**	13.0%-14.4%	12,829	**1,312**	**21.1%**	20.0%-22.5%	6,212	**1,580**	**23.1%**	22.1%-24.1%	6,850
**Filipino**	**3,183**	**21.2%**	20.4%-21.9%	15,048	**567**	**26.3%**	24.5%-28.2%	2,155	**3,333**	**23.5%**	22.8%-24.2%	14,193
**Japanese**	**236**	**16.3%**	14.6%-18.2%	1,444	**362**	**23.6%**	21.6%-25.8%	1,533	**1,660**	**24.7%**	23.5%-25.9%	6,733
**Korean**	**902**	**17.6%**	16.6%-18.7%	5,115	**248**	**24.8%**	22.0%-27.7%	1,002	**788**	**25.5%**	24.0%-27.0%	3,095
**Other Asian**	**711**	**15.5%**	14.4%-16.7%	4,590	**376**	**21.9%**	20.0%-23.9%	1,716	**1,244**	**20.6%**	19.5%-21.7%	6,037
**Southeast Asian**	**685**	**18.3%**	16.8%-19.9%	3,741	**341**	**23.9%**	21.8%-26.2%	1,426	**833**	**22.9%**	21.4%-24.4%	3,645
**Vietnamese**	**1,762**	**17.1%**	16.1%-18.1%	10,327	**743**	**21.6%**	20.2%-23.1%	3,441	**761**	**25.3%**	23.7%-27.0%	3,008
	**Monoracial Native Hawaiian or Pacific Islander**				**Multiracial Asian + Native Hawaiian or Pacific Islander**				**Multiracial Native Hawaiian or Pacific Islander[c]**			
**Native Hawaiian or Pacific Islander[d]**	**624**	**17.3%**	16.1%-18.6%	3,608	**358**	**26.3%**	24.1%-28.7%	1,360	**1,698**	**25.5%**	24.4%-26.6%	6,654

[a] Southeast Asian includes Cambodian, Hmong and Laotian. Survey participants were not asked to describe specific Asian group for the Other Asian category.
[b] Multiethnic Asian refers to identification with more than one Asian ethnic subgroup. This category is not mutually exclusive; thus, a Chinese and Southeast Asian adolescent would be included twice in the orange bars under Chinese and Southeast Asian. See additional notes under d superscript for Native Hawaiian or Pacific Islander.
[c] Multiracial Asian or Native Hawaiian or Pacific Islander refers to identification as Asian or Native Hawaiian or Pacific Islander (but not both) and another racial or ethnic group of American Indian or Alaska Native, Black, Hispanic or Latinx, multiracial, and White.
[d] Native Hawaiian or Pacific Islander categories are as follow: Monoracial Native Hawaiian or Pacific Islander represents Native Hawaiian or Pacific Islander only. Multiracial Asian+ Native Hawaiian or Pacific Islander includes those who identified as both Asian and Native Hawaiian or Pacific Islander. Multiracial Native Hawaiian or Pacific Islander represents those who identified as both Native Hawaiian or Pacific Islander and another racial or ethnic group of American Indian or Alaska Native, Black, Hispanic or Latinx, multiracial, or White.

© 2024 Lui CK et al. *JAMA Network Open.*

eTable 4. Observed and Predicted Suicidal Ideation Prevalence for Asian Subgroups Across Models in Table 2[a]

**Monoethnic Asians**						**Absolute difference between Observed and predicted prevalence**		
	**N**	**Observed**	**Model 1a**	**Model 1b**	**Model 2**	**Model 1a**	**Model 1b**	**Model 2**
Asian Indian only	9,777	13.29%	13.29%	13.29%	13.31%	0.00%	0.00%	0.03%
Chinese only	12,739	13.69%	13.69%	13.69%	13.70%	0.00%	0.00%	0.01%
Filipino only	14,850	21.10%	21.10%	21.10%	21.05%	0.00%	0.00%	0.06%
Japanese only	1,424	16.29%	16.29%	16.29%	16.46%	0.00%	0.00%	0.16%
Korean only	5,079	17.62%	17.62%	17.62%	17.65%	0.00%	0.00%	0.03%
Other Asian only	4,503	15.43%	15.43%	15.43%	15.84%	0.00%	0.00%	0.41%
Southeast Asian only	3,692	18.28%	18.28%	18.28%	17.94%	0.00%	0.00%	0.04%
Vietnamese only	10,221	17.08%	17.08%	17.08%	17.04%	0.00%	0.00%	0.35%
Simple Average						0.00%	0.00%	0.14%
Weighted Average						0.00%	0.00%	0.08%
**Multiethnic Asians**	**N[b]**	**Observed**	**Model 1a**	**Model 1b**	**Model 2**	**Model 1a**	**Model 1b**	**Model 2**
Chinese + Vietnamese	2,214	19.24%	22.37%	21.48%	19.46%	3.13%	2.24%	0.22%
Chinese + Filipino	1,028	24.81%	21.78%	20.91%	23.65%	3.02%	3.89%	1.15%
Chinese + Other Asian	737	19.95%	21.31%	20.45%	17.58%	1.37%	0.51%	2.37%
Chinese + Japanese	504	14.48%	21.11%	20.26%	18.28%	6.63%	5.78%	3.80%
Chinese + Southeast Asian	399	18.80%	22.50%	21.61%	20.40%	3.71%	2.81%	1.60%
Chinese + Korean	327	15.29%	21.06%	20.22%	19.53%	5.77%	4.92%	4.24%
Japanese + Filipino	293	28.67%	21.33%	23.93%	27.60%	7.34%	4.74%	1.06%
Southeast Asian + Vietnamese	221	25.34%	22.79%	25.51%	24.84%	2.55%	0.17%	0.50%
Asian Indian + Other Asian	186	17.20%	21.83%	24.47%	17.78%	4.62%	7.27%	0.57%
Southeast Asian + Vietnamese + Chinese	178	26.40%	22.94%	22.03%	25.00%	3.46%	4.37%	1.40%
Other Multiethnic Asians	2,103	25.96%	21.66%	23.02%	25.52%	4.31%	2.94%	0.44%
Simple Average						4.17%	3.60%	1.58%
Weighted Average						3.78%	3.02%	1.11%

[a] Southeast Asian includes Cambodian, Hmong and Laotian. Survey participants were not asked to describe specific Asian group for the Other Asian category.
[b] Group order is based on sample size.

© 2024 Lui CK et al. *JAMA Network Open.*
