# Supplementary material for: Unmasking Suicidal Ideation for Asian American, Native Hawaiian, and Pacific Islander Youths Via Data Disaggregation
Source: JAMA Netw Open. 2024 Nov 22;7(11):e2446832. doi: 10.1001/jamanetworkopen.2024.46832 (PMC11584931; doi:10.1001/jamanetworkopen.2024.46832)
Supplement: Supplement 2. — Data Sharing Statement [file jamanetwopen-e2446832-s002.pdf]

Data Sharing Statement

Lui. Unmasking Suicidal Ideation for Asian American, Native Hawaiian, and Pacific Islander Youths Via Data Disaggregation. *JAMA Netw Open*. Published November 22, 2024. doi:10.1001/jamanetworkopen.2024.46832

Data
Data available: No

Additional Information
Explanation for why data not available: Data are available upon request from WestEd California Healthy Kids Survey.
